# Efficacy and Safety of IL-4Rα and IL-5/IL-5R Targeted Biologic Therapies in Type 2 Inflammatory Airway Diseases: A Systematic Review and Meta-Analysis

**DOI:** 10.3390/jcm15135004

**Published:** 2026-06-26

**Authors:** Zhuojun Li, Maoyu Jiang, Maiqi Chen, Yehai Liu

**Affiliations:** 1 Department of Otolaryngology, Head and Neck Surgery, The First Affiliated Hospital of Anhui Medical University, 218 Jixi Road, Hefei 230031, China; 2 The First School of Clinical Medicine, Anhui Medical University, 218 Jixi Road, Hefei 230031, China; 3 School of Basic Medicine, Anhui Medical University, 81 Meishan Road, Hefei 230032, China

**Keywords:** allergic airway diseases, biologic therapy, type 2 inflammation, immunotherapy, IL-4R/IL-5 pathway

## Abstract

**Background/Objectives**: Severe asthma and chronic rhinosinusitis with nasal polyps (CRSwNP) frequently coexist and are associated with type 2 inflammation, leading to poor symptom control and high healthcare burden. Biologic therapies targeting IL-4Rα and IL-5/IL-5R have shown efficacy in type 2 inflammatory asthma and CRSwNP, but comprehensive evidence on their efficacy, safety, and research trends is limited. **Methods**: We conducted a systematic review and meta-analysis of randomized controlled trials (RCTs) evaluating dupilumab, mepolizumab, benralizumab, or reslizumab in patients with type 2 inflammatory asthma and/or CRSwNP. Primary outcomes included lung function (FEV1), symptom control (ACQ, SNOT-22, nasal polyp score), and serious adverse events (SAEs). Risk of bias was assessed using the Cochrane RoB 2.0 tool. Publication bias was evaluated with funnel plots and Trim-and-Fill analysis. Bibliometric analysis was performed to identify publication trends and emerging research directions. **Results**: A total of 23 RCTs involving 8758 participants were included. Biologic therapy was not associated with a significant increase in serious adverse events (RR = 1.15, 95% CI: 0.89–1.50). Compared with control treatment, biologics significantly improved FEV1 (MD = 100.67 mL, 95% CI: 65.94–135.40) and ACQ scores (MD = −0.40, 95% CI: −0.54 to −0.25). In patients with CRSwNP and comorbid asthma, biologics also improved SNOT-22 scores (MD = −13.16, 95% CI: −24.85 to −1.47) and nasal polyp scores (MD = −1.31, 95% CI: −1.95 to −0.68). Dupilumab trials showed larger reductions in nasal polyp score than IL-5/IL-5R-targeted trials, although this indirect comparison should be interpreted cautiously. Bibliometric analysis indicated increasing research attention to upstream epithelial targets such as TSLP. **Conclusions**: Both IL-4Rα and IL-5/IL-5R-targeted biologics are effective and well-tolerated in type 2 inflammatory airway diseases. IL-4Rα inhibition shows favorable upper-airway outcomes in CRSwNP with asthma, but head-to-head trials are needed to clarify its comparative efficacy relative to IL-5/IL-5R-targeted therapies. Emerging research directions are shifting toward upstream epithelial alarmin antibodies.

## 1. Introduction

Asthma, allergic rhinitis, and chronic rhinosinusitis with nasal polyps (CRSwNP) are common and frequently coexisting airway diseases, many of which are driven by type 2 inflammatory mechanisms [1]. Shared epithelial–immune pathways, particularly IL-4/IL-13- and IL-5/IL-5R-mediated responses, contribute to eosinophilic inflammation, mucus hypersecretion, tissue remodeling, and recurrent disease exacerbations. Clinically, these conditions are associated with impaired quality of life and substantial healthcare costs [2,3]. Asthma is a heterogeneous condition. Symptoms may persist despite optimal management (inhalation corticosteroids, long-acting beta agonists). Many patients continue to have suboptimal symptom control as well as frequent exacerbations of their disease [4]. The burden is even greater among patients with comorbidities associated with type 2 inflammation, especially CRSwNP [5].

Over the past decade, significant advances have been made in the understanding of immunologic mechanisms underlying allergic airway inflammation. Type 2 immune responses, which involve an interplay of epithelium-immune cell interaction and cytokine pathways, including the IL-4/IL-13 pathway and IL-5/IL-5R pathway, have been shown to be involved. This leads to the development of an eosinophil-rich inflammation, hypersecretion of mucus, and remodelling of involved tissue structures [6,7]. These discoveries have enabled the development and clinical use of targeted biological therapies, including monoclonal antibodies against IL-5 or the IL-5R (e.g., mepolizumab, benralizumab, and reslizumab), and IL-4Rα inhibitors (e.g., dupilumab) [8,9]. These novel agents have changed the management of severe asthma and some other diseases. They have demonstrated significant clinical benefit in patients who have had little or no success with traditional treatment alternatives.

Nevertheless, despite rapid growth in research, conclusive and consistent clinical results remain limited. Although many clinical studies have assessed the efficacy and safety issues of biologics in respiratory allergy, the existing literature seems to be quite scattered and heterogeneous. Individual clinical studies are often limited by small sample sizes, narrow patient populations, and differences in study designs (such as intervention regimens, follow-up durations, and outcome indicators), leading to inconsistent or even contradictory conclusions regarding the therapeutic effects and safety profiles of biological agents [10,11,12]. In addition to uncertainty regarding efficacy, the expanding use of biologic therapies has heightened concerns about long-term safety and tolerability [13]. This meta-analysis therefore aims to synthesize the available evidence, providing additional insights into the efficacy and safety profiles of these treatments.

In recent years, research on the application of immunotherapy in allergic airway diseases has increased substantially, encompassing mechanistic studies, therapeutic efficacy evaluations, and cohort-based investigations, with a growing trend toward interdisciplinary integration. As the volume of literature continues to expand, traditional approaches to literature review have become increasingly insufficient for comprehensively identifying research trends and emerging directions [14]. To address this limitation, the present study conducted a bibliometric analysis based on data retrieved from the Web of Science Core Collection from 2016 to 2025, aiming to characterize research developments in this field and provide insights to inform future therapeutic strategies and individualized management of allergic airway diseases.

In this study, a systematic review and meta-analysis of randomized controlled trials (RCTs) was conducted to evaluate the efficacy and safety of IL-5/IL-5R-targeted biologics and IL-4R pathway inhibitors in patients with asthma and related type 2 inflammatory airway diseases. The bibliometric component was used to complement the quantitative synthesis by mapping research evolution, identifying emerging therapeutic targets, and contextualizing the clinical evidence within the broader development of biologic therapy for type 2 inflammatory airway diseases.

## 2. Materials and Methods

### 2.1. Meta-Analysis and Systematic Review

#### 2.1.1. Registration

The study was designed and reported in accordance with the Preferred Reporting Items for Systematic Reviews and Meta-Analyses (PRISMA) 2020 guidelines [15]. The PRISMA 2020 checklist was used to guide reporting and is provided in the Supplementary Materials (File S1). The study protocol was registered on the International Prospective Register of Systematic Reviews (PROSPERO, CRD420251252256). As this study exclusively involved the secondary analysis of previously published aggregate data and did not involve the recruitment of human participants or access to identifiable patient-level information, institutional ethics review board approval was not required. This systematic review and meta-analysis was conducted to evaluate the safety and efficacy of biologic therapies targeting the IL-5/IL-5 receptor (IL-5/IL-5R) and IL-4 receptor (IL-4R) pathways in patients with asthma and related type 2 inflammatory airway diseases.

#### 2.1.2. Search Strategy and Selection Process

A comprehensive literature search was conducted across PubMed, Embase, and the Cochrane Central Register of Controlled Trials (CENTRAL) from database inception to 1 November 2025, supplemented by a search of the ClinicalTrials.gov registry to capture ongoing or recently completed clinical trials. The search strategy combined Medical Subject Headings (MeSH) terms and free-text keywords related to asthma, biologic therapy, IL-5/IL-5R inhibitors, IL-4R inhibitors, and randomized controlled trials, yielding a total of 1923 records across all databases (PubMed: 1053; Embase: 642; CENTRAL: 194; ClinicalTrials.gov: 34). All retrieved records were imported into EndNote X9 (Clarivate Analytics), and 192 duplicates were identified and removed, leaving 1731 unique records for title and abstract screening. Of the records proceeding to screening, those related to IL-5/IL-5R therapies and IL-4R therapies were tracked separately throughout the selection process (Table S1).

**Figure 1:**
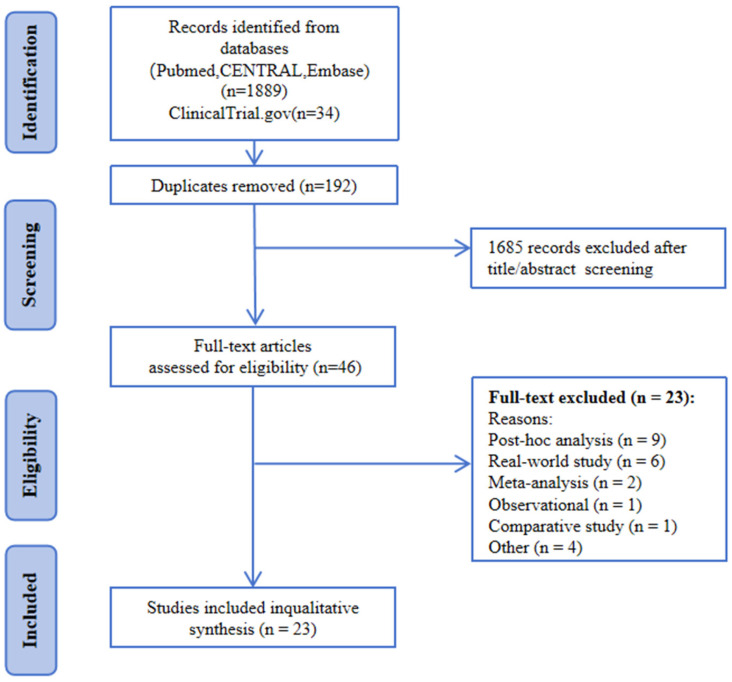
The literature search and screening process for this meta-analysis and systematic review.

Two reviewers independently screened titles and abstracts to identify potentially eligible studies. Only randomized controlled trials (RCTs) were eligible. Non-randomized studies, observational studies, reviews, conference abstracts, case reports, and animal studies were excluded. Full-text articles were subsequently assessed for eligibility according to the predefined inclusion and exclusion criteria. Disagreements between reviewers were resolved through discussion or consultation with a third reviewer. Ultimately, 23 RCTs met the inclusion criteria and were included in the quantitative synthesis (Figure 1).

#### 2.1.3. Inclusion and Exclusion Criteria

The inclusion criteria were formulated in accordance with the PICOS [16] framework, including the following: (1) Population: patients with type 2 inflammatory airway diseases. Eligible populations included: (1) Patients with asthma, allergic rhinitis or severe/eosinophilic asthma, defined by peripheral blood eosinophil counts of ≥150 cells/μL or ≥300 cells/μL; (2) Patients with CRSwNP and CRSwNP accompanied by asthma. (2) Intervention: The interventions of interest were biologic agents targeting type 2 inflammatory pathways, including: (1) IL-5/IL-5R monoclonal antibodies: mepolizumab, benralizumab, and reslizumab; (2) IL-4R monoclonal antibody: dupilumab. (3) Comparator: Eligible studies compared biologic therapy with placebo or an active biologic comparator in addition to standard-of-care asthma treatment, including inhaled corticosteroids (ICS), long-acting β2-agonists (LABA), or other guideline-recommended background therapies. (4) Outcome: The primary efficacy outcome was forced expiratory volume in the first second (FEV1). The primary safety outcome was SAEs. Secondary outcomes included: (1) Change in Asthma Control Questionnaire (ACQ) scores; (2) Change in nasal polyp score; (3) SNOT-22 scores. The following studies were excluded from the meta-analysis: (1) Non-randomized controlled studies (observational studies, case reports, case series); (2) Reviews, meta-analyses, and conference abstracts; (3) Post hoc analyses, subgroup analyses, and summary analyses; (4) Open-label extension studies; (5) Real-world evidence studies; (6) Economic evaluation studies; (7) Studies for which full-text articles could not be retrieved or data were incomplete.

#### 2.1.4. Data Extraction and Risk of Bias Assessment

Data extraction was independently performed by two reviewers using a standardized form. The following data were extracted: study characteristics; participant demographics; intervention details (drug, dosage, route, duration); and outcome data. For dichotomous outcomes (adverse events), event counts and total sample sizes were extracted. For continuous outcomes (FEV1, ACQ, SNOT-22, nasal polyp score), mean change from baseline, standard deviation, and sample size were extracted. When multiple time points were reported, end-of-treatment data were used. When data were missing, corresponding authors were contacted twice at two-week intervals. Unavailable data led to exclusion from the relevant meta-analysis. No imputation was performed. Risk of bias was assessed using the Cochrane RoB 2.0 tool, with each domain rated as low risk, some concerns, or high risk. The robvis package (0.3.0) was used for visualization [17,18].

#### 2.1.5. Statistical Analysis

Meta-analyses were performed using appropriate statistical software. For dichotomous outcomes, pooled effect estimates were calculated as risk ratios (RRs) or odds ratios (ORs) with 95% confidence intervals (CIs). Continuous outcomes were summarized using mean differences (MDs) or standardized mean differences (SMDs), as appropriate. To account for study heterogeneity, the Hartung–Knapp adjustment [19] was applied to calculate more accurate confidence intervals (CIs). Statistical heterogeneity was assessed using the I^2^ statistic [20] and Cochran’s Q test. A random-effects model was applied when substantial heterogeneity was present (I^2^ > 50%); otherwise, a fixed-effects model was used. Publication bias was evaluated using funnel plots and Egger’s linear regression test was used to quantitatively assess funnel plot asymmetry to detect potential publication bias [21]. A *p*-value of <0.05 was considered indicative of significant publication bias. Additionally, the Trim-and-fill method [22] was applied to estimate the number of missing studies and adjust for potential bias caused by unpublished studies.

### 2.2. Bibliometric Study

#### 2.2.1. Data Retrieval and Selection

In this research, the Web of Science Core Collection (WoSCC) was selected as the primary data source. The Web of Science is renowned for its comprehensive coverage of academic literature across various research fields. Scholars highly regard it for its quality and reliability, establishing it as a premier digital archive for academic publications [23]. The data were downloaded on 30 July 2025. The search terms used were as follows: TS = (“Targeted Immunotherapy” OR “Biologic Therapy” OR “Monoclonal Antibodies”) AND (“Asthma*” OR “Wheez*” OR “Bronchial Asthma” OR “Cough* Variant* Asthma” OR “Respiratory Allergy” OR “Allergic Rhinitis” OR “Nasal Allergy”). The time frame was set from 2016 to 2025, selected English as the language, and filtered the results to include only articles and reviews.

Strict inclusion and exclusion criteria were established as follows: (1) studies related to allergic airway diseases and immunotherapy; (2) English-language articles published in peer-reviewed journals within the last ten years; (3) exclusion of studies completely unrelated to airway allergic responses and immunotherapy based on titles and abstracts; (4) removal of duplicate articles or reviews listed in the database. The selection process was jointly conducted by two researchers to ensure the accuracy of the data.

**Figure 2:**
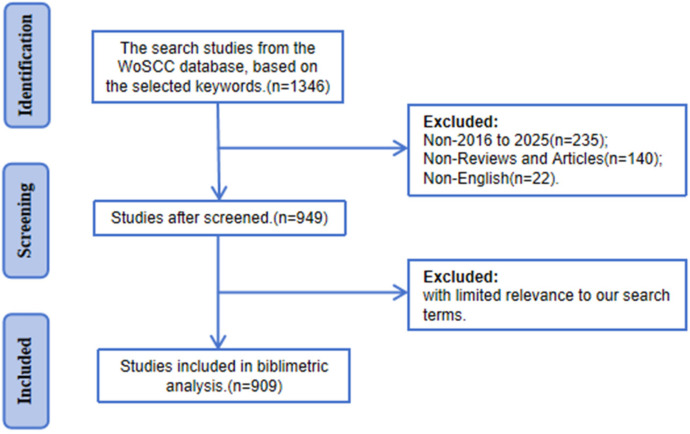
The literature search and screening process for this bibliometric study.

In total, 909 studies were retrieved. Figure 2 shows the literature search and screening process for this study.

#### 2.2.2. Data Analysis

The data obtained from WoSCC were exported in “plain text” format using the “Full Record and Cited References” feature. Widely recognized bibliometric software tools were utilized, including CiteSpace 6.6 and VOSviewer (1.6.20). Additionally, the R package Bibliometrix (5.1.0) was employed for further analysis.

CiteSpace is a widely used bibliometric and scientific knowledge visualization tool [24], which utilizes the LLR algorithm and Kleinberg’s burst detection algorithm for analyzing and visualizing literature data [25]. In this study, it was used to analyze the evolution of keywords and their changes over time, uncover citation relationships between documents, and identify key research themes and emerging trends. Furthermore, it facilitated the understanding of the structure of the research field.

## 3. Results

### 3.1. Meta-Analysis

#### 3.1.1. Quality of Eligible Studies

The risk of bias of the included studies was assessed using the RoB 2.0 tool and classified as low risk, some concerns, or high risk of bias. Overall, bias in outcome measurement was generally judged to be low, and the domains related to deviations from intended interventions and selection of the reported result were also largely assessed as low risk. By contrast, the randomization process and missing outcome data were the main domains contributing to concerns regarding study quality. In several studies, limited information on allocation concealment, handling of incomplete outcome data, or prespecification of outcome analyses resulted in judgments of some concerns or high risk of bias. Overall, the risk-of-bias profile suggests that the main limitations were related to incomplete methodological reporting rather than consistent evidence of substantial internal validity problems (Figure S1). Publication bias was formally assessed for the SAE outcome because this analysis included 10 or more studies. Visual inspection of the funnel plot did not indicate marked asymmetry, and Egger’s test did not suggest significant small-study effects. Therefore, no obvious evidence of publication bias was identified for this outcome (Figure S2).

#### 3.1.2. Safety

**Figure 3:**
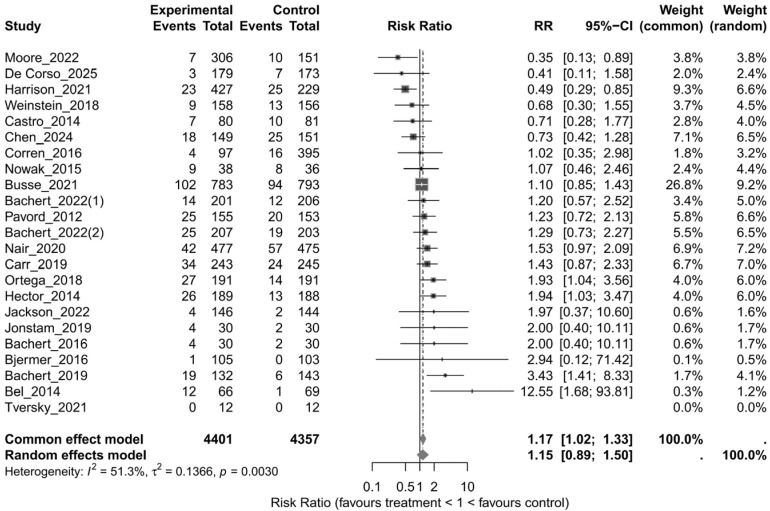
Forest plot of the association between biologic therapy and the risk of SAEs (Moore_2022 [26], De Corso_2025 [27], Harrison_2021 [28], Weinstein_2018 [29], Castro_2014 [30], Chen_2024 [31], Corren_2016 [32], Nowak_2015 [33], Busse_2021 [34], Bachert_2022 (1) [35], Bachert_2022 (2) [36], Pavord_2012 [37], Nair_2020 [38], Carr_2019 [39], Ortega_2018 [40], Hector_2014 [41], Jackson_2022 [42], Jonstam_2019 [43], Bachert_2016 [44], Bachert_2019 [45], Bel_2014 [46], Tversky_2021 [47]. Bjermer_2016 [48]).

**Figure 4:**
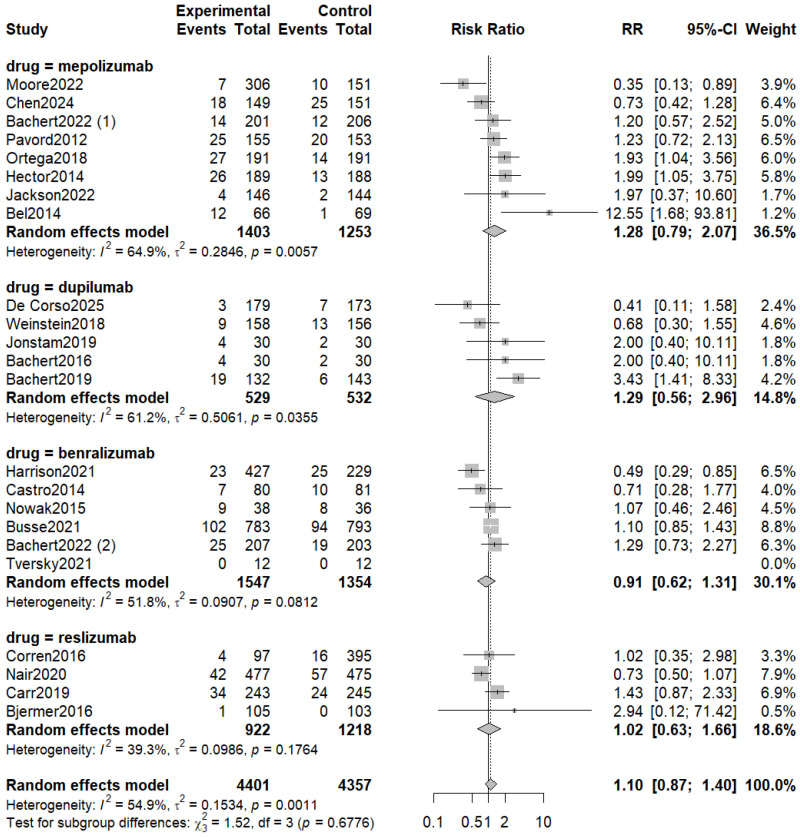
Forest plot of the association between individual biologic agents (benralizumab, mepolizumab, reslizumab, and dupilumab) and the risk of serious adverse events (Moore_2022 [26], De Corso_2025 [27], Harrison_2021 [28], Weinstein_2018 [29], Castro_2014 [30], Chen_2024 [31], Corren_2016 [32], Nowak_2015 [33], Busse_2021 [34], Bachert_2022 (1) [35], Bachert_2022 (2) [36], Pavord_2012 [37], Nair_2020 [38], Carr_2019 [39], Ortega_2018 [40], Hector_2014 [41], Jackson_2022 [42], Jonstam_2019 [43], Bachert_2016 [44], Bachert_2019 [45], Bel_2014 [46], Tversky_2021 [47]. Bjermer_2016 [48]).

A total of 23 studies [26,27,28,29,30,31,32,33,34,35,36,37,38,39,40,41,42,43,44,45,46,47,48] involving 8758 participants were included in the SAE analysis. Overall, biologic therapy was not associated with a significant increase in SAE risk (random-effects model: RR = 1.15, 95% CI: 0.89–1.50), with moderate heterogeneity across studies (I^2^ = 51.3%, *p* = 0.0030) (Figure 3). Given the mechanistic differences among biologics, pathway-stratified analyses were further conducted for IL-5/IL-5R- and IL-4Rα-targeted agents. In the pathway-stratified primary analysis, IL-5/IL-5R-targeted agents were further examined by individual drug classes: benralizumab [28,30,33,34,35,47], mepolizumab [26,31,36,37,40,41,42,46], reslizumab [32,38,39,48], and dupilumab [27,43,44,45] to delineate the SAE risk profile attributable to each biologic within its respective mechanistic category. Benralizumab (RR = 0.91, 95% CI: 0.62 to 1.31), reslizumab (RR = 1.02, 95% CI: 0.63 to 1.66), dupilumab (RR = 1.29, 95% CI: 0.56 to 2.96), and mepolizumab (RR = 1.28, 95% CI: 0.79 to 2.07) did not show a sustained significant increase in the risk of SAEs in the random-effects model (Figure 4).

#### 3.1.3. FEV1

**Figure 5:**
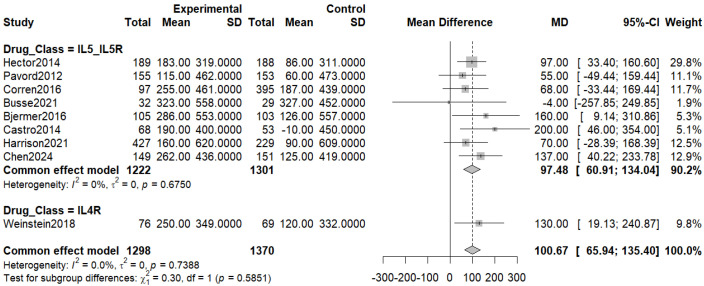
Forest plot of the meta-analysis comparing changes in forced expiratory volume in 1 s (FEV1) between the treatment group and the control group (Harrison_2021 [28], Weinstein_2018 [29], Castro_2014 [30], Chen_2024 [31], Corren_2016 [32], Busse_2021 [34], Pavord_2012 [37], Hector_2014 [41], Bjermer_2016 [48]).

A total of 9 studies involving 1298 patients in the experimental group and 1370 patients in the control group were included in the meta-analysis of change from baseline in FEV1 [28,29,30,31,32,34,37,41,48]. The pooled results showed that, compared with the control group, the experimental group achieved a significant improvement in FEV1, with a mean difference of 100.67 mL (95% CI: 65.94 to 135.4 mL). Heterogeneity was negligible across studies (I^2^ = 0%, τ^2^ = 0, *p* = 0.7388), indicating good consistency of the treatment effect. Most included studies favored the experimental group, and the pooled diamond did not cross the line of no effect, suggesting a clear benefit of biologic therapies in improving lung function (Figure 5). Notably, in addition to studies targeting the IL-5/IL-5R pathway, the included analysis also contained a study targeting the IL-4R pathway. The overall direction of effect remained favorable, suggesting that, beyond IL-5-related mechanisms, IL-4R-targeted therapy may also contribute to improvements in FEV1.

#### 3.1.4. Asthma Control Questionnaire (ACQ)

**Figure 6:**
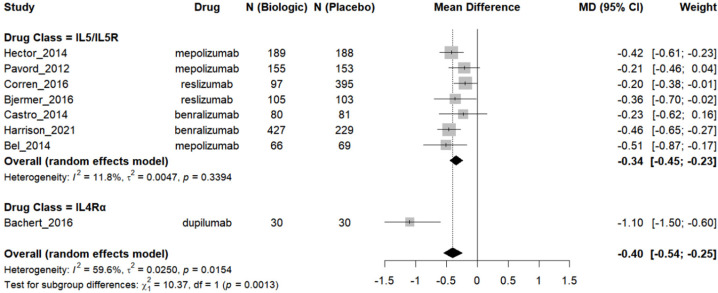
Forest plot of the meta-analysis comparing changes in ACQ scores between the biologic treatment group and the control group (Harrison_2021 [28], Castro_2014 [30], Corren_2016 [32], Pavord_2012 [37], Hector_2014 [41], Bachert_2016 [44], Bel_2014 [46], Bjermer_2016 [48]). The pooled mean difference (MD) was estimated using a random-effects model with Hartung–Knapp adjustment. Negative values favor the biologic group.

Changes in ACQ scores were evaluated as a secondary efficacy endpoint to capture symptom-level treatment response among IL-5/IL-5R-targeted and IL-4R-targeted biologic agents. A total of eight randomized controlled trials [28,30,32,37,41,44,46,48] reporting ACQ change-from-baseline data were included in the pooled analysis. Overall, biologic treatment was associated with a significant improvement in ACQ compared with placebo (MD = −0.40, 95% CI: −0.54 to −0.25) under a random-effects model. Among the included studies, most evidence was derived from IL-5/IL-5R-targeted biologics, including mepolizumab, reslizumab, and benralizumab. In this subgroup, biologic therapy significantly improved ACQ scores compared with placebo (MD = −0.34, 95% CI: −0.45 to −0.23), with low between-study heterogeneity (I^2^ = 11.8%, τ^2^ = 0.0047, *p* = 0.3394), indicating a relatively consistent treatment effect across studies. In addition, one study of the IL-4Rα-targeted biologic dupilumab also showed a favorable effect on ACQ improvement (MD = −1.10, 95% CI: −1.50 to −0.60). Although evidence for this pathway was limited, the direction of effect suggests that, in addition to the more commonly studied IL-5/IL-5R pathway, IL-4Rα-targeted therapy may also contribute to improved asthma symptom control (Figure 6).

#### 3.1.5. SNOT-22

**Figure 7:**
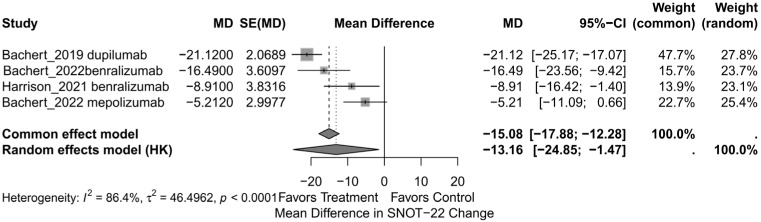
Forest plot of the meta-analysis comparing changes in SNOT-22 scores between the treatment group and the control group (Harrison_2021 [28], Bachert_2022 (1) [35], Bachert_2022 (2) [36], Bachert_2019 [45]. Negative values favor the biologic group.

For patients with CRSwNP and comorbid asthma, the effect of biologic therapies on sinus-related quality of life was evaluated using the 22-item Sino-Nasal Outcome Test (SNOT-22). A total of four eligible studies [28,35,36,45] were included in the meta-analysis, comprising three IL-5/IL-5R-targeted trials (two evaluating benralizumab and one evaluating mepolizumab) and one IL-4Rα antagonist trial (dupilumab). The pooled results demonstrated that biologic therapies significantly improved sinus-related quality of life compared with the control group (MD = −13.16, 95% CI: −24.85 to −1.47, *p* < 0.05), a clinically meaningful reduction in SNOT-22 scores that underscores the therapeutic benefit of targeted biologics in alleviating sinus-related symptoms and enhancing quality of life in this comorbid patient population (Figure 7).

#### 3.1.6. Nasal Polyp Score

**Figure 8:**
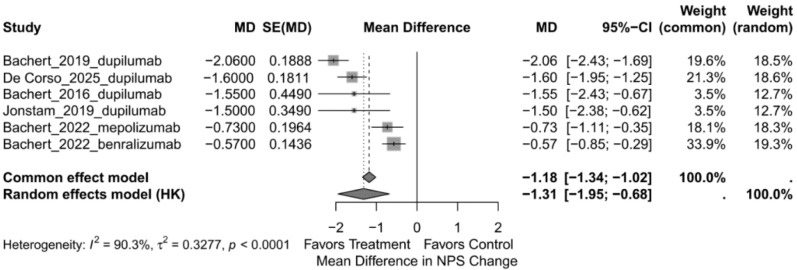
Forest plot of the meta-analysis comparing changes in Nasal Polyp Score (NPS) between the treatment group and the control group (De Corso_2025 [27], Bachert_2022 (1) [35], Bachert_2022 (2) [36], Jonstam_2019 [43], Bachert_2016 [44], Bachert_2019 [45]). Negative values favor the biologic group.

A total of six eligible studies were included in the meta-analysis of change from baseline in nasal polyp score (NPS) [27,35,36,43,44,45], comprising four trials of the IL-4Rα-targeted biologic dupilumab and two trials of IL-5/IL-5R-targeted biologics, including one study of benralizumab and one study of mepolizumab. Substantial between-study heterogeneity was observed (I^2^ = 90.3%, τ^2^ = 0.3277, *p* < 0.0001); therefore, a random-effects model was applied. The pooled analysis showed that biologic therapy significantly reduced NPS compared with the control group, with a mean difference of −1.31 (95% CI: −1.95 to −0.68, *p* < 0.001), indicating a significant improvement in nasal polyp burden in patients with type 2 inflammatory airway disease (Figure 8).

Notably, the four dupilumab trials consistently showed larger reductions in NPS, with individual effect estimates ranging from −2.06 to −1.50, whereas the effect sizes observed in the mepolizumab and benralizumab studies were smaller (MD = −0.73 and −0.57, respectively). This pattern suggests that IL-4Rα-targeted biologics may exert a more pronounced effect on reducing nasal polyp severity than IL-5/IL-5R-targeted therapies. However, given the limited number of studies in each subgroup and the high overall heterogeneity, this finding should be interpreted with caution.

### 3.2. Bibliometric Analysis

#### 3.2.1. Annual Number of Published Papers

**Figure 9:**
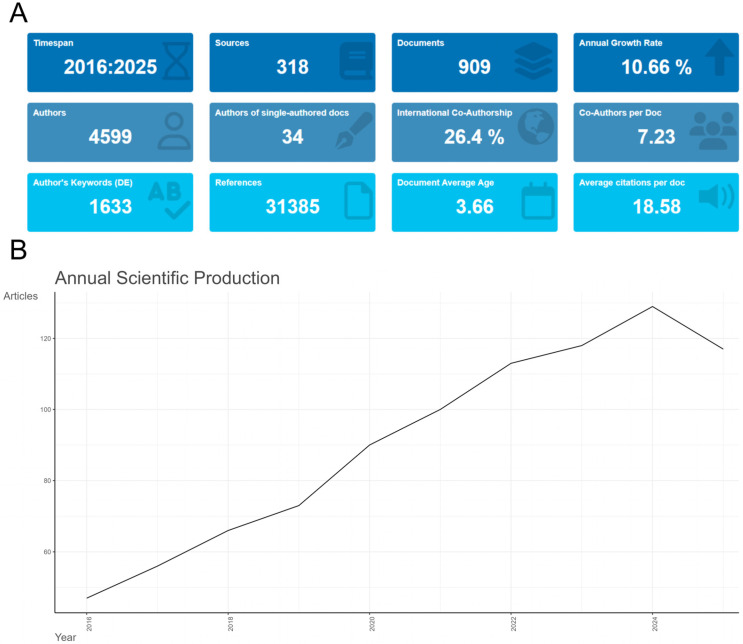
(**A**) Main information description from 2016 to 2025; (**B**) Annual publication trend.

From 2016 to 2025, a total of 909 papers that met the inclusion and exclusion criteria were identified. These papers were co-authored by 4599 authors from 1793 institutions in 65 countries (Figure 9A). An upward trend in the number of publications was observed related to immunotherapy. The field maintained a stable annual growth rate of 10.66%, with more than 100 papers published each year in the past five years. Although previous studies in this field mainly focused on the research of immune regulation mechanisms of allergic airway diseases, in recent years, with the advancement of immune mechanism research, the translational research and clinical application of immunotherapy have also significantly increased. This trend suggests increasing research interest in immunotherapy for allergic airway diseases (Figure 9B).

#### 3.2.2. Keywords Analysis

**Figure 10:**
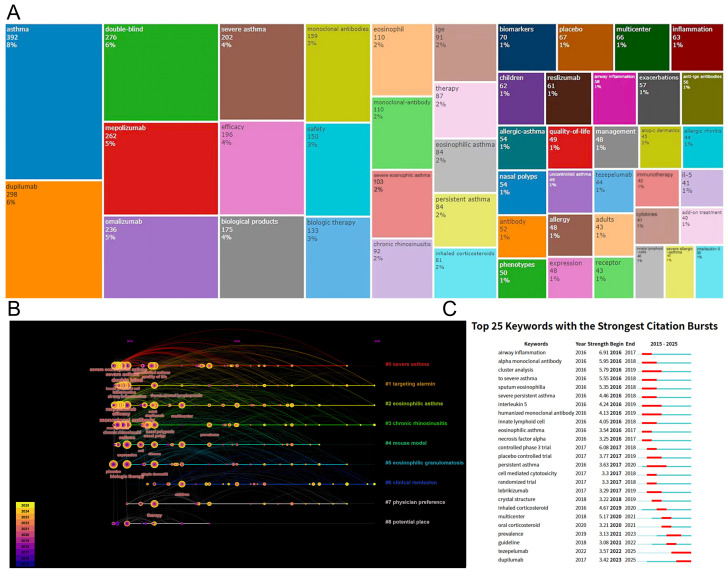
Keyword analysis. (**A**) Treemap visualization of high-frequency keywords; (**B**) CiteSpace keyword timeline map; (**C**) Top 25 keywords with the strongest citation bursts.

A co-occurrence analysis of keywords related to immunotherapy for allergic airway diseases identified 3399 unique keywords. Figure 10A visually presents the key themes and trends in the field from 2016 to 2025, highlighting the core focus on biological therapies for allergic rhinitis and asthma. Among the high-frequency keywords, “Asthma” (392 occurrences), “Dupilumab” (298 occurrences), “Double-blind” (276 occurrences), “Mepolizumab” (262 occurrences), and “Omalizumab” (236 occurrences) are the core focuses. Figure 10B is a keyword timeline generated by CiteSpace, showing the evolution of the themes of biological immunotherapy in this field from 2016 to 2025: “Severe asthma” (#0) and “eosinophilic asthma” (#2) are the core themes throughout, highlighting their importance; early research focused on therapeutic drugs (such as mepolizumab) and clinical trial designs (double-blind, multi-center), and later research expanded to specific scenarios and populations such as animal models (#4 mouse models), pediatric patients, and clinical remission (#6). “Severe asthma” is highly correlated with “chronic rhinosinusitis” (#3) and “eosinophilic asthma”, confirming the concept of upper and lower airway consistency. Figure 10C illustrates the top 25 keywords with the strongest citation bursts between 2015 and 2025. Among them, “airway inflammation” exhibited the highest burst strength (6.91), followed by “monoclonal antibody” (5.95) and “cluster analysis” (5.79). Additionally, “tezepelumab” showed a citation burst beginning in 2022, suggesting its increasing relevance as an emerging therapeutic agent in this field.

#### 3.2.3. Research Distribution Analysis

**Figure 11:**
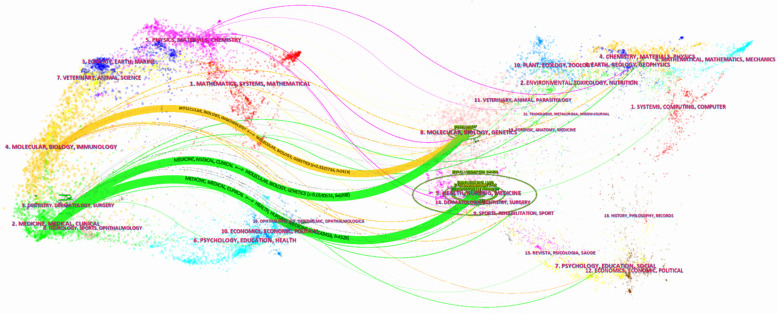
Stacked diagram of research domain collaboration and topic migration.

This study constructed a disciplinary knowledge clustering and flow map based on an interdisciplinary literature dataset, aiming to analyze the research distribution and knowledge associations across various fields. Notably, the medical and life sciences are transmitting a strong knowledge flow to other disciplines, and multiple knowledge exchange channels have been formed between medicine and ecology, psychology, and other disciplines. This suggests that basic sciences may play an important supporting role in interdisciplinary research. In addition, the intersection between “Environmental Sciences” and medicine and life sciences is particularly prominent, reflecting that environmental science and health science are moving towards a deep integration direction, jointly constituting an active interdisciplinary research field. (Figure 11).

## 4. Discussion

This systematic review and meta-analysis synthesized randomized evidence on biologic therapies targeting the IL-4Rα and IL-5/IL-5R pathways in type 2 inflammatory airway diseases. The main findings were that IL-5/IL-5R-targeted biologics significantly improved FEV1, while IL-4Rα-targeted therapy was associated with clinically relevant improvement in sinonasal outcomes. No clear increase in serious adverse events was observed. These results support the clinical use of pathway-specific biologic therapies in appropriately selected patients [49]. In addition, the incorporation of bibliometric analysis offers complementary evidence on research evolution and emerging therapeutic directions. This combined approach may help bridge current randomized evidence with future mechanism-driven and phenotype-guided clinical decision-making.

### 4.1. Therapeutic Efficacy

Overall, biologics targeting the IL-5/IL-5R axis significantly improved FEV1 in patients with severe asthma, indicating meaningful recovery of airflow limitation and lower-airway function. In parallel, IL-5/IL-5R-targeted therapies also demonstrated significant improvement in ACQ scores, suggesting better asthma symptom control and reduced disease burden from the patient perspective. Similarly, therapy targeting IL-4Rα has also been shown to improve asthma symptoms. By simultaneously inhibiting both the IL-4 and IL-13 signaling pathways, IL-4Rα blockade is expected to affect multiple aspects of type 2 inflammation, including immunoglobulin E (IgE) class switching, excessive mucus secretion, epithelial barrier dysfunction, and airway remodeling, thereby helping to enhance asthma control [50,51]. These findings are consistent with previous research results, further confirming the overall clinical effectiveness of biological therapy in severe type 2 asthma [52,53].

In patients with comorbid asthma and CRSwNP, IL-4Rα blockade demonstrated particularly favorable efficacy in upper-airway disease. Consistent with previous reports, dupilumab significantly improved sinonasal symptoms and quality of life in patients with CRSwNP [54]. IL-4R biologics are highly effective in CRSwNP because they target the upstream drivers of type 2 inflammation, thereby reducing eosinophilic inflammation, polyp burden, symptom severity, and recurrence risk [3,51]. Anti-IL-5 monoclonal antibodies likewise demonstrated beneficial effects on CRSwNP. Through depletion of eosinophils, these agents were associated with reductions in polyp size and improvements in olfactory function, with some patients exhibiting clinical benefit within 12 weeks of treatment initiation. These findings indicate that targeted biologic therapy can effectively modulate inflammatory responses in both the upper and lower airways [55]. In our meta-analysis, IL-4Rα-targeted therapy with dupilumab showed greater improvements in both nasal polyp score and SNOT-22 score than IL-5/IL-5R-targeted therapies. However, it should be emphasized that these comparisons were indirect, as no head-to-head trials have directly compared dupilumab with mepolizumab or benralizumab in patients with CRSwNP and comorbid asthma. Therefore, the observed differences in effect size should be interpreted as exploratory and hypothesis-generating rather than as evidence of therapeutic superiority. A potential mechanistic explanation for the more pronounced effects associated with IL-4Rα blockade is its broader immunomodulatory activity: by inhibiting both IL-4 and IL-13 signaling, this approach targets upstream drivers of type 2 inflammation rather than focusing solely on eosinophil depletion, which may be particularly relevant in patients with comorbid asthma [3,55,56,57].

Taken together, our findings support the concept that the efficacy of biologic therapies may vary according to the pattern of airway involvement. In the overall severe asthma population, biologics effectively improved pulmonary function and asthma symptom control, but IL-4Rα-targeted therapy did not demonstrate a clear advantage in FEV1 over other biologic classes. In contrast, among patients with concomitant CRSwNP, IL-4Rα blockade appeared to confer additional benefit in upper-airway disease control. This phenotype-specific therapeutic pattern is consistent with the concept of unified airway disease, while also suggesting that the relative contribution of individual cytokine pathways may differ between the upper and lower airways.

These findings may have important implications for biologic selection in clinical practice. Multiple classes of biologics can provide meaningful benefit in severe asthma, as reflected by improvements in both objective and subjective disease outcomes. However, in patients with prominent sinonasal comorbidity—particularly those with severe polyp burden, persistent nasal obstruction, or impaired olfactory function—IL-4Rα-targeted therapy may provide broader and more comprehensive disease control across both airway compartments.

Given the heterogeneity of type 2 inflammation and the variable response to biologic therapies, the identification of appropriate biomarkers may help optimize biologic selection and improve therapeutic outcomes. Biomarkers such as blood eosinophil count, serum IgE level, fractional exhaled nitric oxide (FeNO), and comorbid CRSwNP status may provide clinically relevant information for predicting treatment responsiveness and guiding individualized therapy. Future studies should therefore focus on biomarker-driven treatment algorithms to facilitate precision medicine approaches in severe type 2 airway disease [58,59].

Future studies should also incorporate clinically meaningful endpoints beyond symptom scores and lung function, including severe exacerbation reduction, oral corticosteroid-sparing effects, and clinical remission, as these outcomes are increasingly important for assessing biologic effectiveness and guiding individualized treatment selection.

Notably, within the IL-5/IL-5R-targeted class, benralizumab has a distinctive afucosylated structure that enables antibody-dependent cellular cytotoxicity (ADCC) through CD16-expressing natural killer cells, potentially exerting immunomodulatory effects beyond eosinophil depletion. Although these agents were analyzed together in the present study according to their shared role in targeting eosinophilic inflammation, IL-5- and IL-5R-targeted therapies should not be considered fully interchangeable at the mechanistic level. Future drug-specific or subgroup analyses may help determine whether these mechanistic differences are associated with clinically relevant differences in treatment response [60,61,62].

### 4.2. Safety and Tolerability

The overall safety profile of biologic therapies was acceptable, and no significant increase in SAEs was observed across biologic classes, including IL-4Rα- and IL-5/IL-5R-targeted therapies. These findings are generally consistent with previous randomized controlled trials and meta-analyses evaluating biologic treatment in severe type 2 airway disease [63,64]. One possible explanation for the favorable safety profile is the relatively selective mechanism of biologic therapies. Unlike systemic immunosuppressive agents, biologics primarily target specific inflammatory pathways involved in type 2 inflammation while largely preserving global immune function. Consequently, pathway-specific inhibition of IL-5, IL-4, or IL-13 signaling may reduce inflammatory burden without substantially increasing the risk of severe systemic adverse effects. In addition, previous studies have shown that in specific cases of severe asthma, the combined use of biologics is safe [65]. Notably, IL-4Rα-targeted therapy demonstrated a safety profile comparable to that of IL-5/IL-5R-targeted biologics, including in analyses involving patients with comorbid CRSwNP. This finding is clinically relevant because patients with severe asthma and upper-airway comorbidities often require prolonged treatment duration and repeated therapeutic interventions. The absence of a significant increase in SAEs supports the feasibility of biologic therapy in patients with complex airway disease phenotypes. However, several issues should be interpreted cautiously. Follow-up duration varied considerably across studies, and long-term real-world safety data remain relatively limited for some biologics. In addition, heterogeneity in SAE definitions and adverse-event reporting among trials may have influenced pooled estimates. Therefore, continued pharmacovigilance and long-term observational studies are still needed to further clarify the safety profile of biologic therapies in broader clinical populations.

### 4.3. Future Directions and Implications

The bibliometric component of this study complements the quantitative synthesis by addressing questions that meta-analysis alone cannot fully answer: which therapeutic targets are emerging, how research priorities evolve over time, and what contextual factors may influence the future clinical application of biologic therapies. Meta-analysis can quantitatively assess the therapeutic effects based on strict trial conditions, while bibliometric mapping can reflect broader scientific priorities and emerging development directions [66]. In this study, the convergence between the bibliometric trends (such as the increasing attention to IL-4R, IL-5/IL-5R, and TSLP) and the clinical efficacy demonstrated by these targets indicates the significance of the current research direction.

Recent bibliometric findings indicate a growing scholarly focus on upstream inflammatory regulators, particularly thymic stromal lymphopoietin (TSLP), reflecting a broader shift in the understanding of severe asthma pathobiology from isolated effector pathways to the epithelial–immune interface. As an epithelial-derived alarmin released in response to allergens, microorganisms, pollutants, and other environmental insults, TSLP occupies an early position in the inflammatory cascade and acts as a key initiator of downstream immune activation. In this context, the growing focus on anti-TSLP therapy is mechanistically plausible. By binding TSLP with high affinity and preventing its interaction with the TSLPR/IL-7Rα receptor complex, tezepelumab interrupts inflammatory signaling at an upstream level, thereby attenuating multiple downstream pathways rather than targeting a single effector component. This feature may partly explain why its therapeutic activity appears to extend beyond conventional type 2-high eosinophilic asthma and remain relevant in patients with lower type 2 inflammatory signatures, such as those with neutrophil-predominant or ILC2-independent disease patterns [67,68]. The expanding research interest identified in our bibliometric analysis therefore not only reflects publication trends, but also mirrors an evolving therapeutic paradigm toward broader coverage of asthma heterogeneity. Given that tezepelumab has already entered clinical practice for severe asthma, future studies should further clarify its long-term safety, durability of benefit across distinct airway inflammatory endotypes, and its optimal positioning relative to existing biologics, including sequential or combination strategies in carefully selected patients.

Furthermore, the close interdisciplinary connection established between medicine and environmental science highlights the growing emphasis on the environmental-immune interaction in allergic airway diseases. Environmental exposure factors, including air pollution, allergens, and climate variations, can reshape the airway immune environment, influence inflammatory phenotypes, and potentially alter the therapeutic response to biological agents [69,70]. For instance, long-term exposure to pollutants can cause some patients to shift from eosinophilic airway inflammation to non-eosinophilic airway inflammation, which directly affects the efficacy of targeted biological agents for specific phenotypes [71]. In this context, targeting epithelial-derived mediators such as TSLP has emerged as a highly promising strategy, as these molecules act as molecular bridges between environmental stimuli and immune activation. Therefore, future research can explore strategies that target multiple upstream mediators simultaneously. In addition to TSLP, other alarmins, such as IL-33, are increasingly recognized as promising upstream therapeutic targets. Monoclonal antibodies that block the IL-33/ST2 pathway, including itepekimab and astegolimab, are currently under clinical investigation. Similar to tezepelumab, these agents may challenge the traditional paradigm of asthma precision therapy, which has largely relied on biomarkers such as blood eosinophil counts, fractional exhaled nitric oxide (FeNO), and immunoglobulin E (IgE) to identify type 2-high phenotypes. By targeting upstream epithelial–immune signaling, anti-alarmin therapies may provide clinical benefit across broader inflammatory phenotypes, including patients whose disease cannot be fully explained by eosinophilic inflammation alone or who respond inadequately to existing biologics targeting IL-4Rα or IL-5/IL-5R. The parallel development of TSLP and IL-33 pathway inhibitors reflects an important shift in therapeutic strategy from targeting single downstream cytokines toward broader upstream modulation of type 2 and non–type 2 inflammatory cascades [72,73,74,75].

## 5. Limitations

This study has several limitations. The number of trials evaluating IL-4Rα-targeted therapy, particularly for sinonasal outcomes, was limited, and heterogeneity existed in some endpoints due to differences in study design, patient populations, and follow-up duration. Additionally, comparisons between IL-4Rα- and IL-5/IL-5R-targeted biologics were based on indirect, cross-study evidence rather than head-to-head trials; therefore, no definitive conclusions regarding the superiority of one biologic class over the other can be drawn. Moreover, several clinically relevant outcomes that are increasingly used to guide biologic selection, including annualized severe exacerbation rate, oral corticosteroid (OCS) sparing effect, and clinical remission, were not included as pooled endpoints in this meta-analysis. This was primarily because of inconsistent definitions, variable reporting across trials, or lack of available data for all included biologic agents. Importantly, subgroup analyses by baseline blood eosinophil counts or other type 2 biomarkers were not feasible because of inconsistent reporting, leaving the influence of eosinophilic status on treatment response unclear. Additionally, long-term safety and real-world effectiveness could not be fully assessed, and evidence on sequential or combination biologic use remains sparse. Future studies with standardized biomarker data and extended follow-up are warranted to guide precision therapy. These limitations should therefore be taken into account when interpreting the findings and applying them to individualized biologic treatment selection in clinical practice.

## 6. Conclusions

In summary, both IL-4Rα- and IL-5/IL-5R-targeted biologics are effective and well-tolerated in type 2 inflammatory airway diseases. IL-4Rα inhibition shows favorable upper-airway outcomes in patients with CRSwNP and comorbid asthma, but head-to-head trials are needed to directly compare its efficacy with IL-5/IL-5R-targeted therapies. Emerging research directions are shifting toward upstream epithelial alarmin antibodies, which may extend therapeutic benefits beyond traditional type-2-high phenotypes. These findings support phenotype-guided biologic selection and highlight the need for direct comparative studies.

## Data Availability

The data that support the findings of this study are available from the corresponding author upon reasonable request.

## Supplementary Materials

The following supporting information can be downloaded at: https://www.mdpi.com/article/10.3390/jcm15135004/s1, Table S1: Search strategy; Table S2: Characteristics of Included Studies; Figure S1: Risk assessment of bias in included studies for the Meta-analysis (Cochrane RoB 2.0 tool); Figure S2: The funnel plot is used to assess publication bias of different outcome indicators in a meta-analysis; File S1: Prisma 2020 checklist.

## Abbreviations

The following abbreviations are used in this manuscript:

FEV1	Forced Expiratory Volume in one second
ACQ	Asthma Control Questionnaire
SNOT-22	22-item Sino-Nasal Outcome Test
CI	Confidence Intervals
OR	Odds Ratio
RR	Risk Ratio
MD	Mean Difference
SAE	Serious Adverse Event
CRSwNP	Chronic Rhinosinusitis with Nasal Polyps
