# Supplementary material for: Efficacy and Safety of IL-4Rα and IL-5/IL-5R Targeted Biologic Therapies in Type 2 Inflammatory Airway Diseases: A Systematic Review and Meta-Analysis
Source: J Clin Med. 2026 Jun 26;15(13):5004. doi: 10.3390/jcm15135004 (PMC13362385; doi:10.3390/jcm15135004)
Supplement: Supplementary file 1 [file jcm-15-05004-s001.zip › jcm-4341294-supplementary.pdf]

Supplement Table S1. Search strategy

Databases	Search strategy
**PubMed**	("Asthma"[Mesh] OR asthma[tiab] OR "severe asthma"[tiab] OR "eosinophilic asthma"[tiab] OR "Rhinitis, Allergic"[Mesh] OR "allergic rhinitis"[tiab] OR "Nasal Polyps"[Mesh] OR "nasal polyp*"[tiab] OR "chronic rhinosinusitis with nasal polyps"[tiab] OR CRSwNP[tiab]) AND (dupilumab[tiab] OR mepolizumab[tiab] OR benralizumab[tiab] OR reslizumab[tiab] OR "IL-5"[tiab] OR "IL-5R"[tiab] OR "IL-5 receptor"[tiab] OR "IL-4R"[tiab] OR "IL-4 receptor"[tiab] OR "IL-4Rα"[tiab]) AND (randomized controlled trial[pt] OR controlled clinical trial[pt] OR randomized[tiab] OR randomised[tiab] OR placebo[tiab] OR trial[tiab])
**Embase**	(asthma:ti,ab OR "severe asthma":ti,ab OR "eosinophilic asthma":ti,ab OR "allergic rhinitis":ti,ab OR "nasal polyp*":ti,ab OR "chronic rhinosinusitis with nasal polyps":ti,ab OR CRSwNP:ti,ab) AND (dupilumab:ti,ab OR mepolizumab:ti,ab OR benralizumab:ti,ab OR reslizumab:ti,ab OR "IL-5":ti,ab OR "IL-5R":ti,ab OR "IL-5 receptor":ti,ab OR "IL-4R":ti,ab OR "IL-4 receptor":ti,ab OR "IL-4Rα":ti,ab) AND (randomized:ti,ab OR randomised:ti,ab OR placebo:ti,ab OR trial:ti,ab)
**CENTRAL**	(asthma OR "severe asthma" OR "eosinophilic asthma" OR "allergic rhinitis" OR "nasal polyp*" OR "chronic rhinosinusitis with nasal polyps" OR CRSwNP) AND (dupilumab OR mepolizumab OR benralizumab OR reslizumab OR "IL-5" OR "IL-5R" OR "IL-5 receptor" OR "IL-4R" OR "IL-4 receptor" OR "IL-4Rα") AND (randomized OR randomised OR placebo OR trial)
**ClinicalTrials.gov**	Condition/disease: asthma OR allergic rhinitis OR chronic rhinosinusitis with nasal polyps OR nasal polyps OR CRSwNP; Other terms: dupilumab OR mepolizumab OR benralizumab OR reslizumab OR IL-5 OR IL-5R OR IL-4R OR IL-4 receptor; Study type: Interventional Studies

Supplement Table S2. Characteristics of Included Studies.

Supplement Table 2. Characteristics of included studies											
**First author**	**Year**	**Population**	**Country/region**	**Study design**	**Intervention**	**Comparator**	**Dose/route**	**Duration (weeks)**	**N (intervention)**	**N (control)**	**Main outcomes**
De Corso	2025	CRSwNP with asthma	Multinational	International, randomized, double-blind, phase 4, head-to-head trial	dupilumab	Active comparator	300 mg SC q2w	24	179	173	SAE, NPS
Chen	2024	Severe eosinophilic asthma	China	Phase III, multicenter, randomized, double-blind, placebo-controlled, parallel-group trial	mepolizumab	Placebo	100 mg SC q4w	52	149	151	SAE, FEV1
Jackson	2022	Asthma	USA	Randomized, double-blind, placebo-controlled, parallel-group trial	mepolizumab	Placebo plus guidelines-based care	100 mg SC q4w	52	146	144	SAE, Exacerbation
Bachert	2022	CRSwNP with asthma	Multinational (11 countries: USA, Canada, Germany, UK, Poland, Hungary, Russia, South Africa, South Korea, Japan, Australia)	Randomized, double-blind, placebo-controlled, parallel-group, phase 3 study	mepolizumab	placebo (plus standard of care: mometasone furoate nasal spray + saline irrigations)	100 mg SC q4w	52	201	206	SAE, SNOT-22, NPS
Bachert	2022	CRSwNP with asthma	phase 3, randomized, double-blind, placebo-controlled, parallel-group trial	Randomized, double-blind, multicenter, parallel-group, phase III study	benralizumab	Placebo	30 mg SC q4w	56	207	203	SAE, SNOT-22, NPS
Moore	2022	Severe eosinophilic asthma	Multinational	Randomised, double-blind, placebo-controlled, parallel-group, multicentre study	mepolizumab	Placebo	100 mg SC q4w	52	306	151	SAE
Busse	2021	Severe eosinophilic asthma	Multinational	Randomised,phase III	benralizumab	Placebo	30 mg SC q4w	108	783	793	SAE, FEV1
Tversky	2021	severe CRSwNP with asthma	USA	Randomized, double-blind, pilot study	benralizumab	Placebo	30 mg SC q4w	20	12	12	SAE
Harrison	2021	CRSwNP with asthma	Multinational	RCT, phase 3b, double-blind, placebo-controlled	benralizumab	Placebo	30 mg SC q4w/q8w	24	427	229	SAE, FEV1, ACQ, SNOT-22
Nair	2020	Severe eosinophilic asthma	Canada, Australia, France,USA	RCT, phase 3, double-blind, placebo-controlled	reslizumab	Placebo	3 mg/kg q4w	52	477	475	SAE
Carr	2019	Severe eosinophilic asthma	Asia, Australia, North America, South America, South Africa, and Europe	RCT, phase 3, double-blind, placebo-controlled	reslizumab	Placebo	3 mg/kg q4w	52	243	245	SAE
Bachert	2019	CRSwNP with asthma	Multinational	RCT, double-blind, placebo-controlled, parallel-	dupilumab	Placebo	300 mg SC q2w	24	132	143	SAE, SNOT-22, NPS
Jonstam	2019	CRSwNP	USA and Europe	RCT, phase 2, double-blind, placebo-controlled	dupilumab	Placebo	300 mg SC q1w	16	30	30	SAE, NPS
Ortega	2018	Severe eosinophilic asthma	Multinational	Randomized, double-blind, placebo-controlled	mepolizumab	Placebo	100 mg SC q4w	32	191	191	SAE
Weinstein	2018	Allergic rhinitis with asthma	Multinational	RCT	dupilumab	Placebo	300 mg SC q2w	24	158	156	SAE, FEV1
Bjermer	2016	Asthma	Multinational	Randomized, double-blind, placebo-controlled, parallel-group, fixed-dose, phase 3 trial	reslizumab	Placebo	3 mg/kg q4w	16	105	104	SAE, ACQ, FEV1
Corren	2016	Asthma	USA	Randomized, double-blind, placebo-controlled, phase 3 trial	reslizumab	Placebo	3 mg/kg q4w	16	97	395	SAE, ACQ, FEV1
Bachert	2016	CRSwNP with asthma	USA, Belgium, Spain, Sweden	Randomized, double-blind, placebo-controlled parallel-group study	dupilumab	Placebo + mometasone furoate nasal spray	300 mg SC q1w	16	30	30	SAE, NPS,ACQ,
Nowak	2015	Asthma	Multinational	Randomized, double-blind, placebo-controlled	benralizumab	Placebo + outpatient management/usual care	3 mg/kg	12	38	36	SAE
Castro	2014	Severe eosinophilic asthma	USA, Canada, Bulgaria, Brazil, Peru, Mexico, Poland, Russia, Argentina, Colombia	Randomized, controlled, double-blind, dose-ranging phase 2b study	benralizumab	Placebo	2 mg,SC	52	80	81	SAE, ACQ, FEV1
Bel	2014	Severe eosinophilic asthma	United Kingdom	Randomized, double-blind, placebo-controlled trial	mepolizumab	Placebo	100 mg SC q4w	52	66	69	SAE, ACQ
Hector	2014	Severe eosinophilic asthma	United Kingdom	Randomized, double-blind, double-dummy, placebo-controlled trial	mepolizumab	Placebo	100 mg SC q4w	4	189	188	SAE, ACQ, FEV1
Pavord	2012	Severe eosinophilic asthma	Multinational	Multicentre, double-blind, placebo-controlled randomised trial	mepolizumab	Placebo	750 mg intravenous	52	155	153	SAE, ACQ, FEV1

SAE, serious adverse events; FEV1, forced expiratory volume in 1 second; ACQ, Asthma Control Questionnaire; SNOT-22, 22-item Sinonasal Outcome Test; NPS, nasal polyp score; CRSwNP, chronic rhinosinusitis with nasal polyps; SC, subcutaneous; q2w, every 2 weeks; q4w, every 4 weeks; q8w, every 8 weeks.

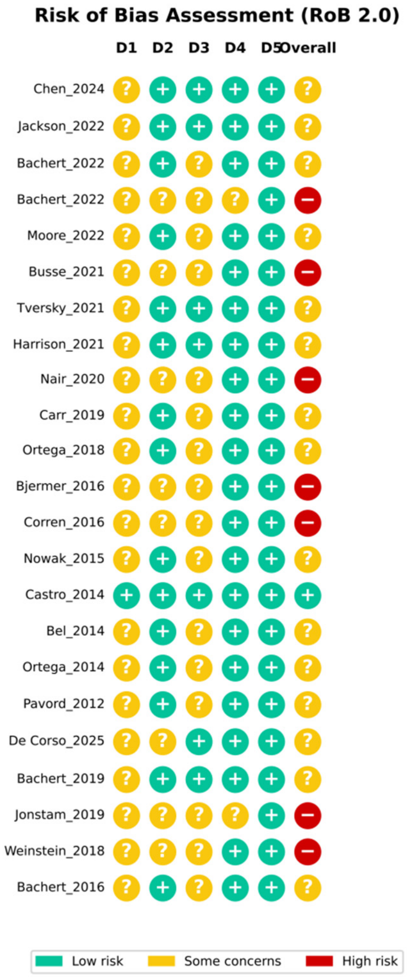

Supplementary Figure S1: Risk assessment of bias in included studies for the Meta-analysis (Cochrane RoB 2.0 tool)

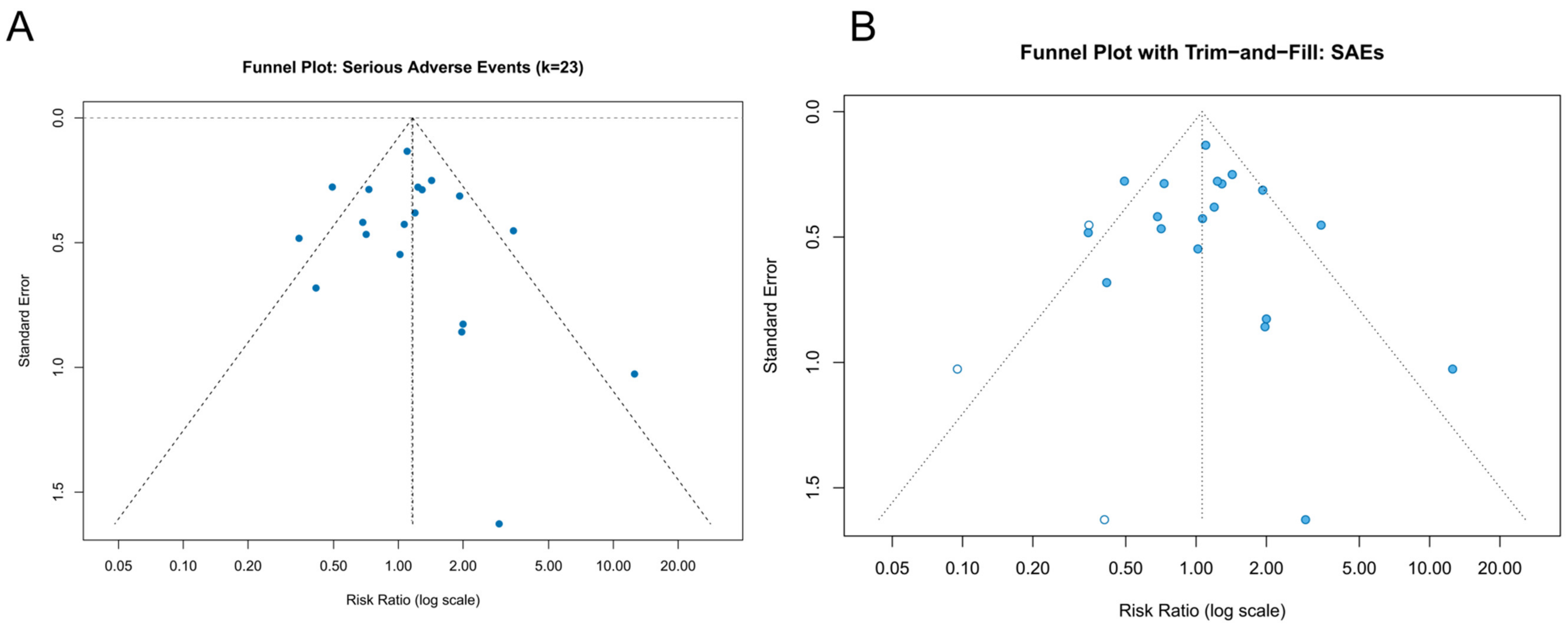

Supplementary Figure S2: The funnel plot is used to assess publication bias of different outcome indicators in a meta-analysis; A: Serious Adverse Events, involving 23 studies, showed a roughly symmetrical distribution on both sides of the central line at each point, suggesting a relatively low risk of publication bias for this outcome indicator; B: The funnel plot after Trim-and-Fill correction shows a more symmetrical overall distribution after correction.

Prisma 2020 checklist

Section and Topic	Item #	Checklist item	Location where item is reported
**TITLE**			
Title	1	Identify the report as a systematic review.	1
**ABSTRACT**			
Abstract	2	See the PRISMA 2020 for Abstracts checklist.	1
**INTRODUCTION**			
Rationale	3	Describe the rationale for the review in the context of existing knowledge.	1-2
Objectives	4	Provide an explicit statement of the objective(s) or question(s) the review addresses.	2
**METHODS**			
Eligibility criteria	5	Specify the inclusion and exclusion criteria for the review and how studies were grouped for the syntheses.	3; Section 2.1.3
Information sources	6	Specify all databases, registers, websites, organisations, reference lists and other sources searched or consulted to identify studies. Specify the date when each source was last searched or consulted.	2; Section 2.1.2
Search strategy	7	Present the full search strategies for all databases, registers and websites, including any filters and limits used.	2; Supplementary Table S1
Selection process	8	Specify the methods used to decide whether a study met the inclusion criteria of the review, including how many reviewers screened each record and each report retrieved, whether they worked independently, and if applicable, details of automation tools used in the process.	2–3; Section 2.1.2; Figure 1
Data collection process	9	Specify the methods used to collect data from reports, including how many reviewers collected data from each report, whether they worked independently, any processes for obtaining or confirming data from study investigators, and if applicable, details of automation tools used in the process.	3; Section 2.1.4
Data items	10a	List and define all outcomes for which data were sought. Specify whether all results that were compatible with each outcome domain in each study were sought (e.g. for all measures, time points, analyses), and if not, the methods used to decide which results to collect.	3; Section 2.1.3
	10b	List and define all other variables for which data were sought (e.g. participant and intervention characteristics, funding sources). Describe any assumptions made about any missing or unclear information.	3; Section 2.1.4; Supplementary Table S2
Study risk of bias assessment	11	Specify the methods used to assess risk of bias in the included studies, including details of the tool(s) used, how many reviewers assessed each study and whether they worked independently, and if applicable, details of automation tools used in the process.	3; Section 2.1.4
Effect measures	12	Specify for each outcome the effect measure(s) (e.g. risk ratio, mean difference) used in the synthesis or presentation of results.	3; Section 2.1.5

Section and Topic	Item #	Checklist item	Location where item is reported
Synthesis methods	13a	Describe the processes used to decide which studies were eligible for each synthesis (e.g. tabulating the study intervention characteristics and comparing against the planned groups for each synthesis (item #5)).	3; Section 2.1.3
	13b	Describe any methods required to prepare the data for presentation or synthesis, such as handling of missing summary statistics, or data conversions.	3; Section 2.1.4
	13c	Describe any methods used to tabulate or visually display results of individual studies and syntheses.	3; Section 2.1.5; Figures 3–8
	13d	Describe any methods used to synthesize results and provide a rationale for the choice(s). If meta-analysis was performed, describe the model(s), method(s) to identify the presence and extent of statistical heterogeneity, and software package(s) used.	3; Section 2.1.5
	13e	Describe any methods used to explore possible causes of heterogeneity among study results (e.g. subgroup analysis, meta-regression).	3; Section 2.1.5
	13f	Describe any sensitivity analyses conducted to assess robustness of the synthesized results.	Not conducted
Reporting bias assessment	14	Describe any methods used to assess risk of bias due to missing results in a synthesis (arising from reporting biases).	3; Section 2.1.5
Certainty assessment	15	Describe any methods used to assess certainty (or confidence) in the body of evidence for an outcome.	Not formally assessed
**RESULTS**			
Study selection	16a	Describe the results of the search and selection process, from the number of records identified in the search to the number of studies included in the review, ideally using a flow diagram.	2–3; Figure 1
	16b	Cite studies that might appear to meet the inclusion criteria, but which were excluded, and explain why they were excluded.	2–3; Figure 1
Study characteristics	17	Cite each included study and present its characteristics.	3–4; Supplementary Table S2
Risk of bias in studies	18	Present assessments of risk of bias for each included study.	4; Supplementary Figure S1
Results of individual studies	19	For all outcomes, present, for each study: (a) summary statistics for each group (where appropriate) and (b) an effect estimate and its precision (e.g. confidence/credible interval), ideally using structured tables or plots.	4–5; Figures 3–8
Results of syntheses	20a	For each synthesis, briefly summarise the characteristics and risk of bias among contributing studies.	4–5; Supplementary Figure S1
	20b	Present results of all statistical syntheses conducted. If meta-analysis was done, present for each the summary estimate and its precision (e.g. confidence/credible interval) and measures of statistical heterogeneity. If comparing groups, describe the direction of the effect.	4–5; Figures 3–8
	20c	Present results of all investigations of possible causes of heterogeneity among study results.	4–5
	20d	Present results of all sensitivity analyses conducted to assess the robustness of the synthesized results.	Not conducted

Section and Topic	Item #	Checklist item	Location where item is reported
Reporting biases	21	Present assessments of risk of bias due to missing results (arising from reporting biases) for each synthesis assessed.	4; Supplementary Figure S2
Certainty of evidence	22	Present assessments of certainty (or confidence) in the body of evidence for each outcome assessed.	Not formally assessed
**DISCUSSION**			
Discussion	23a	Provide a general interpretation of the results in the context of other evidence.	5
	23b	Discuss any limitations of the evidence included in the review.	6
	23c	Discuss any limitations of the review processes used.	6
	23d	Discuss implications of the results for practice, policy, and future research.	5-6
**OTHER INFORMATION**			
Registration and protocol	24a	Provide registration information for the review, including register name and registration number, or state that the review was not registered.	2
	24b	Indicate where the review protocol can be accessed, or state that a protocol was not prepared.	2
	24c	Describe and explain any amendments to information provided at registration or in the protocol.	Not applicable
Support	25	Describe sources of financial or non-financial support for the review, and the role of the funders or sponsors in the review.	6
Competing interests	26	Declare any competing interests of review authors.	6
Availability of data, code and other materials	27	Report which of the following are publicly available and where they can be found: template data collection forms; data extracted from included studies; data used for all analyses; analytic code; any other materials used in the review.	6

From: Page MJ, McKenzie JE, Bossuyt PM, Boutron I, Hoffmann TC, Mulrow CD, et al. The PRISMA 2020 statement: an updated guideline for reporting systematic reviews. BMJ 2021;372:n71. doi: 10.1136/bmj.n71. This work is licensed under CC BY 4.0. To view a copy of this license, visit https://creativecommons.org/licenses/by/4.0/
